# Genetic Diversity of *Cryptosporidium* in Bactrian Camels (*Camelus bactrianus*) in Xinjiang, Northwestern China

**DOI:** 10.3390/pathogens9110946

**Published:** 2020-11-13

**Authors:** Yangwenna Cao, Zhaohui Cui, Qiang Zhou, Bo Jing, Chunyan Xu, Tian Wang, Meng Qi, Longxian Zhang

**Affiliations:** 1 College of Animal Science, Tarim University, Alar 843300, Xinjiang, China; cyangwenna@gmail.com (Y.C.); zqiang1016@gmail.com (Q.Z.); 120050010@taru.edu.cn (B.J.); 120170015@taru.edu.cn (C.X.); 120180029@taru.edu.cn (T.W.); 2 Engineering Laboratory of Tarim Animal Diseases Diagnosis and Control, Xinjiang Production & Construction Corps, Alar 843300, Xinjiang, China; 3 Key Laboratory of Biomarker Based Rapid-detection Technology for Food Safety of Henan Province, Xuchang University, Xuchang 461000, China; czh9008@xcu.edu.cn; 4 College of Veterinary Medicine, Henan Agricultural University, Zhengzhou 450002, China

**Keywords:** *Cryptosporidium*, genotype, Bactrian camels, zoonotic potential, public health

## Abstract

*Cryptosporidium* species are ubiquitous enteric protozoan pathogens of vertebrates distributed worldwide. The purpose of this study was to gain insight into the zoonotic potential and genetic diversity of *Cryptosporidium* spp. in Bactrian camels in Xinjiang, northwestern China. A total of 476 fecal samples were collected from 16 collection sites in Xinjiang and screened for *Cryptosporidium* by PCR. The prevalence of *Cryptosporidium* was 7.6% (36/476). Six *Cryptosporidium* species, *C. andersoni* (*n* = 24), *C. parvum* (*n* = 6), *C. occultus* (*n* = 2), *C. ubiquitum* (*n* = 2), *C. hominis* (*n* = 1), and *C. bovis* (*n* = 1), were identified based on sequence analysis of the small subunit (SSU) rRNA gene. Sequence analysis of the *gp60* gene identified six *C. parvum* isolates as subtypes, such as If-like-A15G2 (*n* = 5) and IIdA15G1 (*n* = 1), two *C. ubiquitum* isolates, such as subtype XIIa (*n* = 2), and one *C. hominis* isolate, such as Ixias IkA19G1 (*n* = 1). This is the first report of *C. parvum*, *C. hominis*, *C. ubiquitum*, and *C. occultus* in Bactrian camels in China. These results indicated that the Bactrian camel may be an important reservoir for zoonotic *Cryptosporidium* spp. and these infections may be a public health threat in this region.

## 1. Introduction

*Cryptosporidium* is a significant cause of diarrheal disease worldwide, with broad host ranges and the ability to infect all vertebrate groups, including humans [1]. As commonly seen, the transmission of enteric pathogens through contaminated surface water, such as *Cryptosporidium* spp. potentially cause large outbreaks of water- and food-borne infections in human populations [2]. Cryptosporidiosis is a global disease and is considered an important opportunistic disease in immunocompromised patients due to its high association with mortality in AIDS patients [3].

Characterization of pathogens at the species or genotype level is mandatory when assessing the potential sources of infection, pathogen load in animals, the environment, transmission routes in human populations, and public health relevance [1,4]. Currently, *Cryptosporidium* genotyping is mostly based on PCR and sequencing of the small subunit (SSU) rRNA gene, which has revealed no less than 40 valid species and more than 70 genotypes of *Cryptosporidium* in humans and animals [1,5,6]. Humans infected by approximately 20 *Cryptosporidium* species and genotypes, with *C. hominis* and *C. parvum*, are responsible for the highest proportion (~90%) of human *Cryptosporidium* infections globally [7]; nevertheless, several primarily animal pathogens, such as *C. meleagridis*, *C. felis*, *C. canis*, and *C. cuniculus* are less commonly found in humans [7].

In northwestern China, Bactrian camels (*Camelus bactrianus*) represent the major livestock species, especially in Xinjiang Uygur Autonomous Region (hereinafter referred to as Xinjiang), because they are well adapted to desert and semi-desert areas and provide milk, meat, and camel hair. *Cryptosporidium* infection of camel calves resulted in diarrhea and debility, while infected adult camels showed no symptoms [8]. Camels infected with *Cryptosporidium* have been reported in many countries, such as the United States, Australia, Czech Republic, Algeria, Iran, Egypt, and China [9,10,11,12,13,14,15,16,17,18,19,20]. However, compared with other livestock animals, information on prevalence, species, genotype, and zoonotic potential of *Cryptosporidium* spp. in Bactrian camels is still limited in China. 

**Figure 1:**
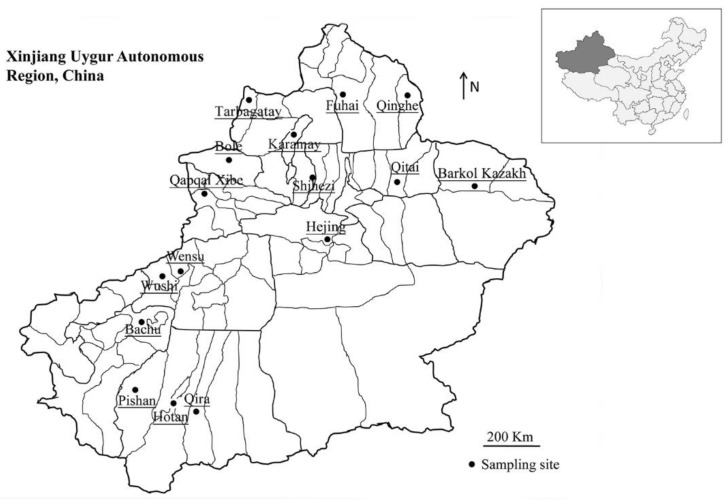
Bactrian camels fecal sampling locations in Xinjiang, northwestern China. No copyright permission was required. The figure was designed with the software ArcGIS 10.2. The map has been originally modified and assembled according to permission and attribution guidelines of the National Geomatics Center of China (http://www.ngcc.cn).

The main focus of the current study was to investigate the prevalence of *Cryptosporidium* and identify the species and subtypes of Bactrian camels in Xinjiang, China (Figure 1). The data will contribute to an improved understanding of *Cryptosporidium* spp. in Bactrian camels and assessment of their zoonotic potential.

## 2. Results

### 2.1. Occurrence of Cryptosporidium

All fecal samples were screened for *Cryptosporidium* by nested PCR targeting of the SSU rRNA gene. In total, 36 samples were *Cryptosporidium*-positive, resulting in an overall infection rate of 7.6% (36/476). In total, 11 of 16 Bactrian camel herds tested contained individuals positive for *Cryptosporidium* spp., and the infection rate at the different collection sites ranged from 0–33.3%; the highest infection rate was observed in Qapqal Xibe County (Table 1).

### 2.2. Cryptosporidium Species and Subtypes

Six species were detected from the 36 *Cryptosporidium*-positive samples. *C. andersoni* (*n* = 24) was the predominant species, followed by *C. parvum* (*n* = 6), *C. ubiquitum* (*n* = 2), *C. occultus* (*n* = 2), *C. hominis* (*n* = 1), and *C. bovis* (*n* = 1) (Table 1). The six *C. parvum*-positive samples were identified once again by restriction fragment length polymorphism (RFLP) analysis, and no mixed infections were found. Phylogenetic analysis revealed that all *C. andersoni* sequences were identical to the GenBank sequence KX710084, derived from Bactrian camels in China. Two types of sequences were identified from the six *C. parvum* isolates: *C. parvum* type 1 (*n* = 5) and *C. parvum* type 2 (*n* = 1) were identical to Genbank sequences KX259139 and KX259140, respectively, derived from deer in China. The two sequences of *C. occultus* were identical to sequence MK982467, derived from calves in Bangladesh. Moreover, the sequence of *C. hominis* was identical to sequence KU200955, derived from horses, while the sequence of *C. bovis* was identical to sequence MF074602, derived from dairy cattle in China. Two sequence types were identified in the two *C. ubiquitum* isolates: *C. ubiquitum* type 1 was identical to sequence KT235697, derived from goats in China, while *C. ubiquitum* type 2 represented a new sequence, bearing two single-nucleotide polymorphism (SNP) deletions at positions 485 and 486 and one SNP substitution at position 298 (A to G), compared with KT235697.

**Figure 2:**
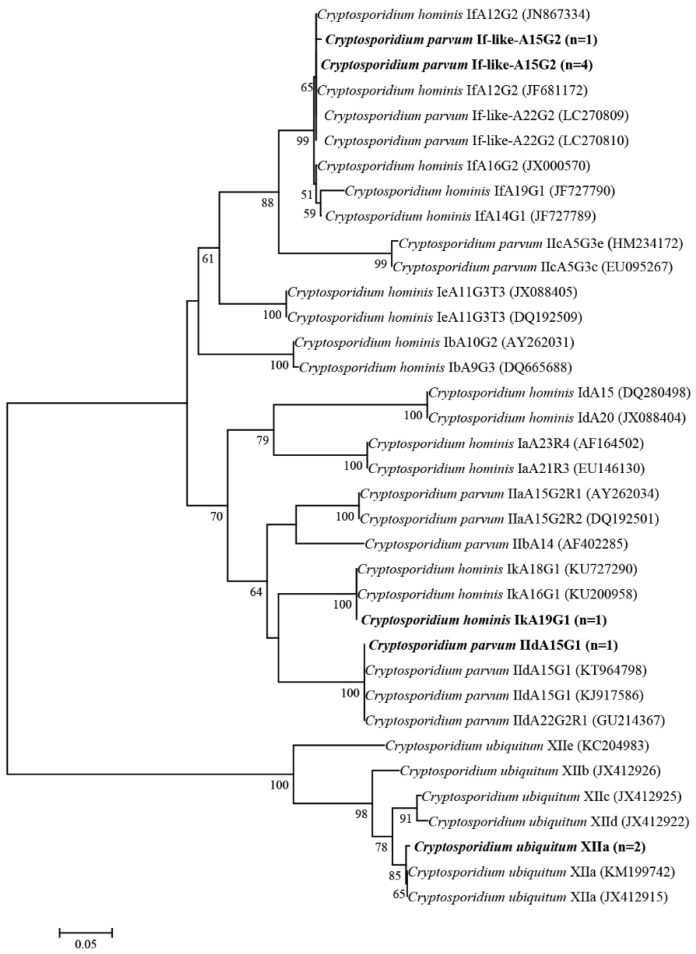
Phylogenetic relationships between *Cryptosporidium* spp. partial *gp60* sequences obtained in this study and sequences retrieved from the GenBank database. Phylogenetic trees were constructed using neighbor-joining methods based on genetic distance, calculated using the Kimura two-parameter model implemented in MEGA 7.0. Bootstrap values >50% from 1000 replicates are indicated at each node. Isolates from this study are shown in bold.

Sequence and phylogenetic analysis of the *gp60* gene revealed two subtypes present in the five *C. parvum* isolates: If-like-A15G2 (*n* = 5) and IIdA15G1 (*n* = 1). The sequence of If-like-A15G2 was similar to an isolate derived from a Swedish patient infected in South Africa (JN867334), except for the copy number differences in the trinucleotide repeat (A15G2 versus A12G2). The sequence of IIdA15G1 was identical to sequence KT964798, derived from dairy cattle in China. The single *C. hominis* isolate was subtyped as IkA19G1 and was similar to sequence KU727290, derived from an infected human patient in Sweden (A19G1 versus A18G2). The two new sequences of *C. ubiquitum* identified were identical to one another and subtyped to family XIIa. All of the subtype sequences, If-like, IId, Ik, and XIIa, clustered with published sequences, If-like, IId, Ik, and XIIa, respectively (Figure 2).

## 3. Discussion

Camels are well known as the ships of the desert and are famous as the beasts of the burden. Camels provide wool, milk, meat, leather, and even dung as fuel for the people in many semi-arid and arid zones, mainly in Africa and Asia [21]. Currently, camel husbandry has been transforming from nomadism to intensive production, resulting in the increase of the total population of camels, with an estimated global population of 35 million [21]. This intensive farming practice of camels has been posing an increased risk for zoonotic disease transmission to humans [22]. Many zoonotic parasites are reported to be transmitted from camels to humans globally [21]. However, there is scarce knowledge regarding camel parasites and their zoonotic importance in China. In this study, the overall *Cryptosporidium* prevalence was 7.6% (36/476), and six species of *Cryptosporidium* (*C. andersoni*, *C. parvum*, *C. hominis*, *C. ubiquitim*, *C. occultus*, and *C. bovis*) were identified, which indicated the genetic diversity of *Cryptosporidium* in Bactrian camels from Xinjiang, China.

From previously published studies, *C. andersoni*, *C. parvum*, *C. muris*, *C. bovis*, *Cryptosporidium* rat genotype IV, and camel genotype have been detected in camels [19]. Among them, only two species of *Cryptosporidium* have been reported in China, namely *C. andersoni* and *C. bovis* [10,12,13,14]. In the present study, both *C. andersoni* and *C. bovis* were identified, and *C. andersoni* was the dominant genotype detected in Bactrian camels. Although *C. andersoni* and *C. bovis* are commonly seen in calves and sheep, *C. andersoni* has also been found in several human cases [23,24]. 

Perhaps unsurprisingly, the most important zoonotic *Cryptosporidium* species., *C. parvum*, was previously reported in Dromedary camels in Algeria, Australia, and Egypt [11,15,19]. According to sequence analysis of the *gp60* gene, two subtypes of *C. parvum* were identified: IIdA15G1 and If-like-A15G2. In China, *C. parvum* isolates, including IIdA14G1, IIdA15G1, IIdA17G1, IIdA18G1, and IIdA19G1, mostly belong to the IId subtype family in goats, humans, cattle, donkeys, horses, rodents, monkeys, Golden takins, and yaks [1]. Previous studies have shown that IIdA15G1 was the predominant subtype in dairy calves and yaks in northwestern China [25,26]. In the present study, IIdA15G1 was identified in Bactrian camels in northwestern China, further confirming the dominance of the IIdA15G1 subtype in western China.

A unique *C. parvum* subtype If-like-A15G2 isolate was identified in Bactrian camels in the current research, which was similar to a previously observed If-like-A22G2 isolate found in Dromedary camels in Algeria [11]. Moreover, subtypes IIaA17G2R1, IIaA15G1R1, and IIdA19G1 were also identified in Dromedary camels in Australia and Egypt [15,19]. The *gp60* gene is highly polymorphic and can be used to categorize *C. parvum* and *C. hominis* into multiple subtypes according to nucleotide sequence differences [27]. However, it seems that *gp60* polymorphisms are ineffective for *C. parvum* subtype identification in camels. In the phylogenetic analysis of *gp60* sequences, *C. parvum* If-like genetically related to the *C. hominis* If subfamily and all If and If-like sequences formed a large clade (Figure 2). More extensive genetic characterization is needed to improve our understanding of the genetic similarity between *C. parvum* and *C. hominis* within the *gp60* gene.

Using *gp60* sequence analysis, *C. hominis* subtype IkA19G1 appeared to belong to subfamily Ik. Family Ik is commonly found in horses and donkeys [28,29] and has been also isolated from patients in Sweden and squirrel monkeys in China [30,31]. This is the first report of *C. hominis* in camels. Further studies should be carried out to expand the biological characterization of *C. hominis* subtype family Ik due to its potential for zoonotic transmission. 

*C. ubiquitum* has a worldwide distribution, and six subtypes/families (XIIa–XIIf) have been identified [32]. Among these subtypes, subtype XIIa has been commonly observed in humans and a wide range of animals, especially domestic and wild ruminants [33,34]. In this study, *C. ubiquitum* and subtype XIIa were detected in Bactrian camels, which indicated that *C. ubiquitum* has a broad host range and high significance for zoonotic infection in this region. *C. occultus*, previously described as *Cryptosporidium* suis-like, was recognized as a valid species in 2018 and has been identified in cattle, yaks, alpacas, and wild rats in China [35,36,37,38]. Moreover, cases of human infection with *C. occultus* have also been found in Canada, China, and the UK [39,40,41]. The present study is the first report of *C. ubiquitum* and *C. occultus* in camels. Further studies into the epidemiology of *Cryptosporidium* infection in both human and livestock is essential.

## 4. Materials and Methods

### 4.1. Ethics Approval

The study was designed and conducted in accordance with the Guide for the Care and Use of Laboratory Animals of the Ministry of Health in China. The Research Ethics Committee of Tarim University critically reviewed this research protocol (approval no. ECTU 2016-0007) and then cleared it for performing. Finally, before fecal sample collection from Bactrian camels, appropriate permission was obtained from the farm owners. 

### 4.2. Sample Collection

In total 476 fresh fecal samples were collected randomly from Bactrian camels grouped into 16 herds located at 16 collection sites in Xinjiang, from July 2016 to September 2019 (Figure 1). Each herd contained between 30 and 300 Bactrian camels. The Bactrian camels were free grazing in desert and semi-desert areas, so their age could not be accurately divided. In some of these areas, cattle, sheep, and horses in pastures also grazed freely. The Bactrian camels had access to pastures or areas where cattle, sheep, and horses had grazed. For each animal, the fresh fecal sample was collected from the ground immediately after defecation, and only one sample was collected per animal into a plastic container that was marked with the sample number and site. After shipping to the laboratory in a cool condition, the fecal samples were stored at 4 °C prior to DNA extraction.

### 4.3. DNA Extraction and PCR Amplification

The E.Z.N.A.^®^ Stool DNA kit (Omega Biotek Inc., Norcross, GA, USA) was used to extract the total DNA from 200 mg of each precipitated sample, according to the manufacturer’s recommendations. PCR analysis of the small subunit (SSU) rRNA gene was employed to screen the infection of *Cryptosporidium* spp. in fecal samples in Bactrian camels [42]. Furthermore, PCR amplification and subsequent sequencing of the 60-kDa glycoprotein (*gp60*) gene were used to subtype *C. parvum*, *C. ubiquitum*, and *C. hominis* [32,43]. The PCR reactions for the SSU rRNA and *gp60* genes conducted in 25 μL reaction mixtures consisted of 12.5 μL of 2 × EasyTaq PCR SuperMix (TransGen Biotech, Beijing, China), 0.3 μM of each primer, 1 μL of DNA sample, and 10.9 μL double-distilled water. *C. parvum* was also determined using restriction fragment length polymorphism (RFLP) analysis, as previously described [44].

### 4.4. Sequencing and Phylogenetic Analysis

Positive PCR amplicons were two-directionally sequenced at GENEWIZ (Suzhou, China). Sequences were assembled and edited using DNAstar Lasergene Editseq 7.1.0 (http://www.dnastar.com/), and reference sequences downloaded from the GenBank database were compared to determine the genotype and subtype of *Cryptosporidium* using ClustalX 2.1 (http://www.clustal.org/). The established nomenclature system was used in the naming subtype of *C. parvum* [11]. Phylogenetic analyses were conducted using neighbor-joining methods based on the Kimura-2 parameter model in MEGA 7.0 (http://www.megasoftware.net/). Seven presentative nucleotide sequences obtained in this study were submitted in the GenBank database (https://www.ncbi.nlm.nih.gov/) under the accession numbers: MH442993–MH442996, MT703861, MT703862, and MT724047.

## 5. Conclusions

Ultimately, Bactrian camels were infected with diverse *Cryptosporidium* species in Xinjiang, northwestern China. Most of these microorganisms have been reported in humans, showing their potential public health relevance and requiring the attention of public health authorities. More molecular studies may be helpful to assess the importance and genetic diversity of *Cryptosporidium* in this region.

## Figures and Tables

**Table 1:** Occurrence of *Cryptosporidium* species/subtypes in Bactrian camels in Xinjiang, China.

Collection Sites	N/T (%) [95% Cl]	*Cryptosporidium* Species/Subtypes (No.)
Barkol Kazakh	0/58 (0)	None
Qinghe	0/57 (0)	None
Qitai	2/18 (11.1) [0–27.2]	*C. andersoni* (1); *C. hominis* (1)/IkA19G1 (1)
Fuhai	1/26 (3.8) [0–11.8]	*C. parvum* (1)/If-like-A15G2 (1)
Karamay	3/45 (6.7) [0–14.2]	*C. andersoni* (3)
Shihezi	5/60 (8.3) [1.1–15.5]	*C. parvum* (5)/If-like-A15G2 (4), IIdA15G1 (1)
Hejing	5/16 (31.3) [5.7–56.8]	*C. andersoni* (5)
Tarbagatay	3/16 (18.8) [0–40.2]	*C. andersoni* (3)
Bole	0/61 (0)	None
Qapqal Xibe	4/12 (33.3) [2–64.6]	*C. andersoni* (4)
Wensu	1/24 (4.2) [0–12.8]	*C. bovis* (1)
Wushi	2/10 (20.0) [0–50.2]	*C. andersoni* (2)
Bachu	0/17 (0)	None
Pishan	3/17 (17.6) [0–37.9]	*C. andersoni* (1); *C. ubiquitum* (2)/XIIa (2)
Hotan	0/17 (0)	None
Qira	7/22 (31.8) [10.7–53.0]	*C. andersoni* (5); *C. occultus* (2)
Total	36/476 (7.6) [5.2–9.9]	*C. andersoni* (24); *C. bovis* (1); *C. occultus* (2); *C. parvum* (6)/If-like-A15G2 (5), IIdA15G1 (1); *C. hominis* (1)/IkA19G1 (1); *C. ubiquitum* (2)/XIIa (2)

N = Number of positives for *Cryptosporidium*; T = Total analyzed samples.

## References

[1] Xiao L., Feng Y. (2017). Molecular epidemiologic tools for waterborne pathogens *Cryptosporidium* spp. and *Giardia duodenalis*. Food Waterborne Parasitol..

[2] Efstratiou A., Ongerth J.E., Karanis P. (2017). Waterborne transmission of protozoan parasites: Review of worldwide outbreaks—An update 2011–2016. Water Res..

[3] Wang R.J., Li J.Q., Chen Y.C., Zhang L.X., Xiao L.H. (2018). Widespread occurrence of *Cryptosporidium* infections in patients with HIV/AIDS: Epidemiology, clinical feature, diagnosis, and therapy. Acta Trop..

[4] Khan A., Shaik J.S., Grigg M.E. (2018). Genomics and molecular epidemiology of *Cryptosporidium* species. Acta Trop..

[5] Liang N., Wu Y., Sun M., Chang Y., Lin X., Yu L., Hu S., Zhang X., Zheng S., Cui Z. (2019). Molecular epidemiology of *Cryptosporidium* spp. in dairy cattle in Guangdong Province, South China. Parasitology.

[6] Ryan U., Fayer R., Xiao L. (2014). *Cryptosporidium* species in humans and animals: Current understanding and research needs. Parasitology.

[7] Feng Y., Ryan U.M., Xiao L. (2018). Genetic diversity and population structure of *Cryptosporidium*. Trends Parasitol..

[8] Sazmand A., Joachim A. (2017). Parasitic diseases of camels in Iran (1931–2017)—A literature review. Parasite.

[9] Sazmand A., Rasooli A., Nouri M., Hamidinejat H., Hekmatimoghaddam S. (2012). Prevalence of *Cryptosporidium* spp. in Camels and involved people in Yazd Province, Iran. Iran. J. Parasitol..

[10] Zhang Q., Zhang Z., Ai S., Wang X., Zhang R., Duan Z. (2019). *Cryptosporidium* spp., *Enterocytozoon bieneusi*, and *Giardia duodenalis* from animal sources in the Qinghai-Tibetan Plateau Area (QTPA) in China. Comp. Immunol. Microbiol. Infect. Dis..

[11] Baroudi D., Zhang H., Amer S., Khelef D., Roellig D.M., Wang Y., Feng Y., Xiao L. (2018). Divergent *Cryptosporidium parvum* subtype and *Enterocytozoon bieneusi* genotypes in dromedary camels in Algeria. Parasitol. Res..

[12] Gu Y., Wang X., Zhou C., Li P., Xu Q., Zhao C., Liu W., Xu W. (2016). Investigation on *Cryptosporidium* infections in wild animals in zoo in Anhui Province. J. Zoo Wildl. Med..

[13] Liu X., Zhou X., Zhong Z., Deng J., Chen W., Cao S., Fu H., Zuo Z., Hu Y., Peng G. (2014). Multilocus genotype and subtype analysis of *Cryptosporidium andersoni* derived from a Bactrian camel (Camelus bactrianus) in China. Parasitol. Res..

[14] Wang R., Zhang L., Ning C., Feng Y., Jian F., Xiao L., Lu B., Ai W., Dong H. (2008). Multilocus phylogenetic analysis of *Cryptosporidium andersoni* (Apicomplexa) isolated from a bactrian camel (*Camelus bactrianus*) in China. Parasitol. Res..

[15] Zahedi A., Lee G.K.C., Greay T.L., Walsh A.L., Blignaut D.J.C., Ryan U.M. (2018). First report of *Cryptosporidium parvum* in a dromedary camel calf from Western Australia. Acta Parasitol..

[16] Xiao L., Escalante L., Yang C., Sulaiman I., Escalante A.A., Montali R.J., Fayer R., Lal A.A. (1999). Phylogenetic analysis of *Cryptosporidium* parasites based on the small-subunit rRNA gene locus. Appl. Environ. Microbiol..

[17] Kvác M., Sak B., Kvetonová D., Ditrich O., Hofmannová L., Modrý D., Vítovec J., Xiao L. (2008). Infectivity, pathogenicity, and genetic characteristics of mammalian gastric *Cryptosporidium* spp. in domestic ruminants. Vet. Parasitol..

[18] Morgan U.M., Xiao L., Monis P., Sulaiman I., Pavlasek I., Blagburn B., Olson M., Upton S.J., Khramtsov N.V., Lal A. (2000). Molecular and phylogenetic analysis of *Cryptosporidium muris* from various hosts. Parasitology.

[19] El-Alfy E.S., Abu-Elwafa S., Abbas I., Al-Araby M., Al-Kappany Y., Umeda K., Nishikawa Y. (2019). Molecular screening approach to identify protozoan and trichostrongylid parasites infecting one-humped camels (*Camelus dromedarius*). Acta Trop..

[20] Abdelwahab A., Abdelmaogood S. (2011). Identification of *Cryptosporidium* species infecting camels (*Camelus dromedarius*) in Egypt. J. Am. Sci..

[21] Sazmand A., Joachim A., Otranto D. (2019). Zoonotic parasites of dromedary camels: So important, so ignored. Parasites Vectors.

[22] Zhu S., Zimmerman D., Deem L. (2019). A review of zoonotic pathogens of Dromedary Camels. Ecohealth.

[23] Leoni F., Amar C., Nichols G., Pedraza-Díaz S., McLauchlin J. (2006). Genetic analysis of *Cryptosporidium* from 2414 humans with diarrhoea in England between 1985 and 2000. J. Med. Microbiol..

[24] Jiang Y., Ren J., Yuan Z., Liu A., Zhao H., Liu H., Chu L., Pan W., Cao J., Lin Y. (2014). *Cryptosporidium andersoni* as a novel predominant *Cryptosporidium* species in outpatients with diarrhea in Jiangsu Province, China. BMC Infect. Dis..

[25] Cui Z., Wang R., Huang J., Wang H., Zhao J., Luo N., Li J., Zhang Z., Zhang L. (2014). Cryptosporidiosis caused by *Cryptosporidium parvum* subtype IIdA15G1 at a dairy farm in Northwestern China. Parasites Vectors.

[26] Qi M., Wang H., Jing B., Wang D., Wang R., Zhang L. (2015). Occurrence and molecular identification of *Cryptosporidium* spp. in dairy calves in Xinjiang, Northwestern China. Vet. Parasitol..

[27] Thompson R.C.A., Ash A. (2016). Molecular epidemiology of *Giardia* and *Cryptosporidium* infections. Infect. Genet. Evol..

[28] Jian F., Liu A., Wang R., Zhang S., Qi M., Zhao W., Shi Y., Wang J., Wei J., Zhang L. (2016). Common occurrence of *Cryptosporidium hominis* in horses and donkeys. Infect. Genet. Evol..

[29] Laatamna A.E., Wagnerová P., Sak B., Květoňová D., Xiao L., Rost M., McEvoy J., Saadi A.R., Aissi M., Kváč M. (2015). Microsporidia and *Cryptosporidium* in horses and donkeys in Algeria: Detection of a novel *Cryptosporidium hominis* subtype family (Ik) in a horse. Vet. Parasitol..

[30] Lebbad M., Winiecka-Krusnell J., Insulander M., Beser J. (2018). Molecular characterization and epidemiological investigation of *Cryptosporidium hominis* IkA18G1 and *C*. *hominis* monkey genotype IiA17, two unusual subtypes diagnosed in Swedish patients. Exp. Parasitol..

[31] Liu X., Xie N., Li W., Zhou Z., Zhong Z., Shen L., Cao S., Yu X., Hu Y., Chen W. (2015). Emergence of *Cryptosporidium hominis* monkey genotype II and novel subtype family Ik in the Squirrel Monkey (*Saimiri sciureus*) in China. PLoS ONE.

[32] Li N., Xiao L., Alderisio K., Elwin K., Cebelinski E., Chalmers R., Santin M., Fayer R., Kvac M., Ryan U. (2014). Subtyping *Cryptosporidium ubiquitum*,a zoonotic pathogen emerging in humans. Emerg. Infect. Dis..

[33] Huang J., Zhang Z., Zhang Y., Yang Y., Zhao J., Wang R., Jian F., Ning C., Zhang W., Zhang L. (2018). Prevalence and molecular characterization of *Cryptosporidium* spp. and *Giardia duodenalis* in deer in Henan and Jilin, China. Parasites Vectors.

[34] Majeed Q.A.H., El-Azazy O.M.E., Abdou N.M.I., Al-Aal Z.A., El-Kabbany A.I., Tahrani L.M.A., AlAzemi M.S., Wang Y., Feng Y., Xiao L. (2018). Epidemiological observations on cryptosporidiosis and molecular characterization of *Cryptosporidium* spp. in sheep and goats in Kuwait. Parasitol. Res..

[35] Kváč M., Vlnatá G., Ježková J., Horčičková M., Konečný R., Hlásková L., McEvoy J., Sak B. (2018). *Cryptosporidium occultus* sp. n. (*Apicomplexa*: *Cryptosporidiidae*) in rats. Eur. J. Protistol..

[36] Li F., Zhang Z., Hu S., Zhao W., Zhao J., Kváč M., Guo Y., Li N., Feng Y., Xiao L. (2020). Common occurrence of divergent *Cryptosporidium* species and *Cryptosporidium parvum* subtypes in farmed bamboo rats (*Rhizomys sinensis*). Parasites Vectors.

[37] Ma J., Li P., Zhao X., Xu H., Wu W., Wang Y., Guo Y., Wang L., Feng Y., Xiao L. (2015). Occurrence and molecular characterization of *Cryptosporidium* spp. and *Enterocytozoon bieneusi* in dairy cattle, beef cattle and water buffaloes in China. Vet. Parasitol..

[38] Zhang Q., Li J., Li Z., Xu C., Hou M., Qi M. (2020). Molecular identification of *Cryptosporidium* spp. in alpacas (*Vicugna pacos*) in China. Int. J. Parasitol. Parasites Wildl..

[39] Xu N., Liu H., Jiang Y., Yin J., Yuan Z., Shen Y., Cao J. (2020). First report of *Cryptosporidium viatorum* and *Cryptosporidium occultus* in humans in China, and of the unique novel *C. viatorum* subtype XVaA3h. BMC Infect. Dis..

[40] Ong C.S., Eisler D.L., Alikhani A., Fung V.W., Tomblin J., Bowie W.R., Isaac-Renton J.L. (2002). Novel *Cryptosporidium* genotypes in sporadic cryptosporidiosis cases: First report of human infections with a cervine genotype. Emerg. Infect. Dis..

[41] Robinson G., Chalmers R.M., Stapleton C., Palmer S.R., Watkins J., Francis C., Kay D. (2011). A whole water catchment approach to investigating the origin and distribution of *Cryptosporidium* species. J. Appl. Microbiol..

[42] Jiang J., Alderisio K.A., Xiao L. (2005). Distribution of *Cryptosporidium* genotypes in storm event water samples from three watersheds in New York. Appl. Environ. Microbiol..

[43] Alves M., Xiao L., Sulaiman I., Lal A.A., Matos O., Antunes F. (2003). Subgenotype analysis of *Cryptosporidium* isolates from humans, cattle, and zoo ruminants in Portugal. J. Clin. Microbiol..

[44] Xiao L., Morgan U.M., Limor J., Escalante A., Arrowood M., Shulaw W., Thompson R.C., Fayer R., Lal A.A. (1999). Genetic diversity within *Cryptosporidium parvum* and related *Cryptosporidium* species. Appl. Environ. Microbiol..

